# Supplementary material for: Assessment of the Content of Glycoalkaloids in Potato Snacks Made from Colored Potatoes, Resulting from the Action of Organic Acids and Thermal Processing
Source: Foods. 2024 May 29;13(11):1712. doi: 10.3390/foods13111712 (PMC11172196; doi:10.3390/foods13111712)
Supplement: Supplementary file 1 [file foods-13-01712-s001.zip › foods-3018934-supplementary.pdf]

Figure S1. Photo of potato tubers of the Mulberry Beauty variety with red flesh and the Double Fun variety with purple flesh, including the size of the tubers (own photos)

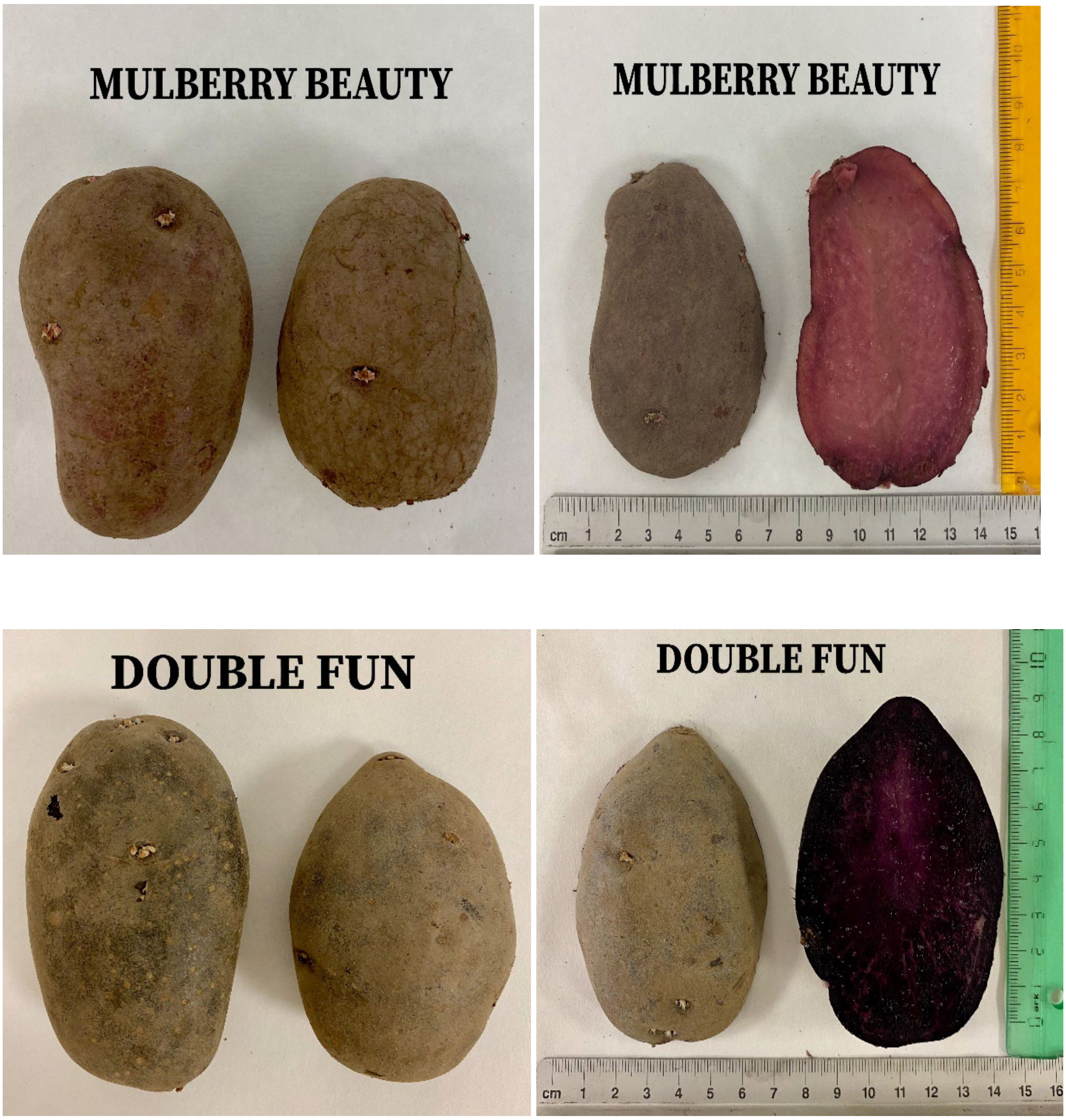

Table S1. The content of glycoalkaloids in samples of pellet snacks obtained from dried potatoes of two varieties, depending on the type of organic acid and the method of expanding used for their production

Potato variety	Expanding method	Type of acid	α- solanine	α- chaconine	TGA
				[mg·100 g^{-1}]	
	Frying	Control	0.14±0.11h	0.21±0.07l	0.35±0.09i
**Mulberry**		Citric acid	0.08±0.12i	0.23±0.09l	0.31±0.10i
**Beauty**		Lactic acid	0.09±0.20i	0.22±0.15l	0.31±0.17i
		Ascorbic acid	0.14±0.14h	0.31±0.11i	0.45±0.12h
**(MB)**		Malic acid	0.16±0.18g	0.25±0.13k	0.41±0.18h
		Tartaric acid	0.15±0.21g	0.27±0.28j	0.42±0.20h
	Microwaving	Control	0.48±0.18ab	0.85±0.08b	1.33±0.12b
		Citric acid	0.46±0.16b	0.84±0.07bc	1.30±0.11c
		Lactic acid	0.54±0.09a	0.84±0.15bc	1.38±0.12b
		Ascorbic acid	0.50±0.11b	0.76±0.19d	1.26±0.15c
		Malic acid	0.40±0.10d	0.57±0.16f	0.97±0.13f
		Tartaric acid	0.54±0.09a	0.82±0.12c	1.36±0.10b
**Double**	Frying	Control	0.14±0.10h	0.27±0.11j	0.41±0.10h
**Fun**		Citric acid	0.18±0.15f	0.39±0.16h	0.57±0.15g
		Lactic acid	0.16±0.21g	0.29±0.13j	0.45±0.17h
**(DF)**		Ascorbic acid	0.24±0.07e	0.38±0.17h	0.62±0.12g
		Malic acid	0.16±0.22f	0.34±0.16i	0.50±0.20gh
		Tartaric acid	0.17±0.11f	0.42±0.14g	0.59±0.12g
	Microwaving	Control	0.44±0.09bc	0.74±0.16d	1.18±0.12d
		Citric acid	0.41±0.09c	0.66±0.12e	1.07±0.10e
		Lactic acid	0.47±0.14b	0.82±0.11c	1.29±0.12c
		Ascorbic acid	0.45±0.17b	0.87±0.16b	1.32±0.15b
		Malic acid	0.37±0.08d	0.67±0.19e	1.04±0.14e
		Tartaric acid	0.52±0.08a	1.04±0.20a	1.56±0.14a

(± SD) – standard deviation; (*n* = 6), a-j - values followed by the same letter, within the same column, were not significantly different ($p > 0.05$), according to Duncan's least significant difference test

Table S2. The content of glycoalkaloids in samples of French fries obtained from two varieties, depending on the type of organic acid used in their production and the method of their preparing for eating

**Potato variety**	**Preparing method**	**Type of acid**	**α- solanine**	**α- chaconine [mg·100 g^{-1}]**	**TGA**
	Frying	Control	0.61±0.11a	2.44±0.15a	3.05±0.13a
**Mulberry**		Citric acid	0.35±0.09b	1.94±0.23b	2.29±0.12c
**Beauty**		Lactic acid	0.34±0.12b	1.25±0.14f	1.59±0.13f
		Ascorbic acid	0.31±0.14c	1.91±0.16b	2.22±0.15c
**(MB)**		Malic acid	0.12±0.08e	1.40±0.18f	1.52±0.12f
		Tartaric acid	0.07±0.07g	1.19±0.19g	1.26±0.11g
	Baking	Control	0.08±0.20f	0.78±0.12j	0.86±0.17j
		Citric acid	0.06±0.15g	0.69±0.16j	0.75±0.15j
		Lactic acid	0.05±0.23h	0.69±0.16j	0.74±0.20j
		Ascorbic acid	0.06±0.17g	1.13±0.09h	1.19±0.12h
		Malic acid	0.09±0.11f	1.27±0.07f	1.36±0.09g
		Tartaric acid	0.09±0.09f	1.03±0.02i	1.12±0.05i
**Double**	Frying	Control	0.07±0.08g	0.75±0.14j	0.82±0.12j
**Fun**		Citric acid	0.11±0.14e	1.27±0.09f	1.38±0.12g
		Lactic acid	0.13±0.18e	1.60±0.08d	1.73±0.12e
**(DF)**		Ascorbic acid	0.04±0.19h	1.30±0.10f	1.34±0.15g
		Malic acid	0.10±0.18f	1.37±0.14f	1.47±0.16fg
		Tartaric acid	0.18±0.11d	1.75±0.16c	1.93±0.14d
	Baking	Control	0.04±0.19h	0.95±0.16i	0.99±0.15i
		Citric acid	0.07±0.21g	0.96±0.19i	1.03±0.16i
		Lactic acid	0.14±0.16e	1.51±0.13e	1.65±0.14e
		Ascorbic acid	0.10±0.19f	2.36±0.14a	2.46±0.15b
		Malic acid	0.05±0.11h	1.57±0.11d	1.62±0.11e
		Tartaric acid	0.07±0.13g	1.97±0.07b	2.04±0.10d

(± SD) – standard deviation (n = 6), a-j -values followed by the same letter, within the same column, were not significantly different ($p > 0.05$), according to Duncan´s least significant difference test
